# The Small GTPase Rap1b: A Bidirectional Regulator of Platelet Adhesion Receptors

**DOI:** 10.1155/2012/412089

**Published:** 2012-06-14

**Authors:** Gianni Francesco Guidetti, Mauro Torti

**Affiliations:** Department of Biology and Biotechnology, Division of Biochemistry, University of Pavia, Via Bassi 21, 27100 Pavia, Italy

## Abstract

Integrins and other families of cell adhesion receptors are responsible for platelet adhesion and aggregation, which are essential steps for physiological haemostasis, as well as for the development of thrombosis. The modulation of platelet adhesive properties is the result of a complex pattern of inside-out and outside-in signaling pathways, in which the members of the Rap family of small GTPases are bidirectionally involved. This paper focuses on the regulation of the main Rap GTPase expressed in circulating platelets, Rap1b, downstream of adhesion receptors, and summarizes the most recent achievements in the investigation of the function of this protein as regulator of platelet adhesion and thrombus formation.

## 1. Introduction

The adhesion of circulating blood platelets to the subendothelial matrix exposed upon vessel wall injury represents the initial event of the haemostatic process required to limit hemorrhage. Platelets express several membrane receptors specific for all the major adhesive ligands of the vascular extracellular matrix [1]. Among these, collagen is probably the most important subendothelial matrix component involved in thrombus formation, and platelet adhesion to collagen is associated with a complex pattern of activatory signaling pathways. Integrin *α*_2_ *β*_1_ and glycoprotein VI (GPVI) are the two main platelet receptors for collagen and, in the rheological conditions of low shear rates, typically present in large veins and venules, are sufficient to mediate firm platelet adhesion. At high shear rates, characteristic of small arteries and stenotic vessels, platelets are unable to efficiently interact to exposed collagen fibers, and in these conditions adhesion is preceded by platelet tethering and rolling on the site of injury. This process is mediated by the membrane GPIb-IX-V complex, a platelet-specific receptor for the multimeric glycoprotein von Willebrand factor (VWF). At high shear stress, circulating VWF rapidly interacts with exposed collagen fibers and undergoes a conformational change that allows the interaction with the GPIb-XI-V complex, decelerating platelets and favoring the subsequent stable adhesion mediated by other platelet receptors [2].

The interaction of platelet adhesion receptors with subendothelial matrix components stimulates an intricate pattern of signal transduction pathways, that trigger spreading, secretion of soluble proaggregating molecules, thromboxane A_2_ (TxA_2_) synthesis and release, and phosphatydilserine exposure. These events recruit and activate additional circulating platelets to initiate a process of cell aggregation, that generates a rapidly growing thrombus at the site of damaged vessel wall.

The stability of platelet adhesion and of the subsequent thrombus formation is reinforced by autocrine stimulation by the released soluble agonists, in particular ADP and TxA_2_, and by thrombin produced through the coagulation cascade. These agonists stimulate specific G-protein-coupled receptors (GPCRs) expressed on platelet surface and typically lead to the activation of phospholipase C (PLC) *β* isoforms, that release diacylglycerol (DAG) and inositol trisphosphate (IP_3_). IP_3_ mediates Ca^2+^ release from intracellular stores, whereas DAG stimulates several effectors containing the DAG-regulated C1 domain, such as classical and novel protein kinase C isoforms [3]. Concomitantly, other signaling molecules, including phosphatidylinositol 3 kinases (PI3Ks), protein tyrosine and serine/threonin kinases, and Ras-like small GTPases, are activated and contribute to promote full platelet activation. These events eventually promote platelet aggregation and thrombus formation, which are supported by the stimulation of integrin *α*_IIb_ *β*_3_, that is converted into a high-affinity state for fibrinogen, whose binding mediates interaction of adjacent cells promoting thrombus growth [2, 4, 5].

Several signaling pathways evoked upon platelet adhesion require the intervention of one or more Ras-like small GTPases, that operate as molecular switches by cycling between an inactive state bound to GDP and an active state bound to GTP through the action of specific guanine nucleotide exchange factors (GEFs) and GTPase-activating proteins (GAPs) [6]. Platelets express several Ras-like GTPase, including Ras [7, 8], Ral [9], Rho [10], Rac [9] and, Cdc42 [11], but are particularly rich of members of the Rap subfamily of GTPases [12]. This paper will focus on platelet Rap GTPases to highlight recent insights into the mechanism of activation and recruitment upon stimulation of the major platelet adhesion receptors.

## 2. Rap GTPases in Platelets

It is now well established that Rap GTPases are involved in several cell functions, including growth, proliferation, cell-cell contact, and adhesion [13]. The Rap family consists of five members: two Rap1 proteins (Rap1a and Rap1b) that share 90% sequence homology and three Rap2 proteins (Rap2a, Rap2b, and Rap2c) which are about 70% homologous to Rap1 [14–16]. An important difference between the two subfamilies is that Rap2 proteins typically display a lower sensitivity to GAPs, promoting a prolonged Rap2 signaling compared to that of Rap1. Although regulated by the same set of GEFs and GAPs, Rap1 and Rap2 can be involved in distinct processes, as shown in cell types different from platelets [17, 18], suggesting that they may operate through different effectors. The biochemical basis of these different effects has not been thoroughly investigated; however it might also be related to the differences in posttranslational modifications and subcellular localization of Rap1 and Rap2 isoforms [19].

 Rap1b is the most abundant GTPase in platelets and represents up to the 0.1% of total proteins, but platelets also contain Rap2b, which, however, is about ten times less abundant than Rap1b. Very low levels of Rap1a and Rap2a have been detected in platelets, whereas Rap2c is not present [12, 16, 20]. Probably because of these different levels of expression, only a limited number of studies have addressed the biochemistry and function of Rap2b in platelets [19, 21–24], while the majority of the investigations have focused on Rap1b.

In platelets, the most abundant and functionally relevant RapGEF is certainly the Ca^2+^- and DAG-regulated CalDAG-GEFI, which represents the key regulator of Rap GTPases activation downstream of PLC activation [25–28]. The expression of low levels of other Rap1GEFs, as PDZ-GEF and CalDAG-GEFIII, has also been reported, but in some cases their presence remains controversial [29, 30]. The only GAP specific for Rap GTPases identified in platelets is Rap1GAP2, whereas Rap1GAP1, SPA-1, and E6TP1 have not been found [29]. It has been shown that Rap1GAP2 is able to associate with 14-3-3 and to modulate cell adhesion when overexpressed in HeLa cells [31]; however the importance of this regulator in platelet function is still unknown.

## 3. Rap GTPases as Regulators of Platelet Integrins and Integrin-Mediated Functions: Insights from Rap1b and CalDAG-GEFI Knockout Mice

The role of Rap GTPases, and in particular of Rap1, in the regulation of cell adhesion is well documented by a number of observations in different cell types [32–35]. Rap GTPases participate to the conversion of integrins into a high-affinity state for their ligands, that in turn allows cells to interact, in a controlled fashion, with other cells and with extracellular matrix components. The ability of integrins to bind their ligands is regulated by inside-out signaling pathways that originate inside the cell and are then transmitted to the extracellular ligand-binding domain of the receptor [36].

Integrin-mediated platelet functions include adhesion, aggregation, and thrombus formation, and thus it appears reasonable for Rap1b to be involved in these responses. In this context, important information has been collected upon the generation of the genetically modified mice that do not express either Rap1b, or the main Rap1GEF present in platelets, CalDAG-GEFI. Rap1b knockout mice display a prolonged tail bleeding time and a marked protection from platelet-dependent arterial thrombosis, demonstrating an essential role of this GTPase in both haemostasis and thrombosis [37]. The importance of Rap1b activation in thrombus formation is also confirmed by analysis of CalDAG-GEFI knockout mice. Although the clotting parameters are normal, CalDAG-GEFI knockout mice display strong defects in haemostasis assessed by bleeding tail analysis [26]. In addition, platelets in whole blood collected from CalDAG-GEFI knockout mice fail to form thrombi when perfused over a fibrillar collagen surface both at low and high shear rates [26, 38]. Moreover, the lack of CalDAG-GEFI is also associated with defective *in vivo *thrombosis, that is virtually abolished in arteries and strongly reduced in venules [38]. These alterations of the haemostatic and thrombotic functions of platelets, which are driven by both cell-matrix and cell-cell adhesion, are indicative of a role for Rap1b in the regulation on platelet integrin function. Platelets express at least five different integrins (*α*_IIb_ *β*_3_, *α*_2_ *β*_1_, *α*_5_ *β*_1_, *α*_6_ *β*_1_, and *α*_v_ *β*_3_) and in particular integrins *α*_IIb_ *β*_3_ and *α*_2_ *β*_1_ play a predominant role in platelet adhesion and activation [2]. However, integrins *α*_5_ *β*_1_ and *α*_6_ *β*_1_ are required to mediate shear-resistant adhesion [39], whereas the function of integrin *α*_v_ *β*_3_ is less understood.

A number of “*ex vivo*” studies on human and murine platelets have demonstrated that Rap1b plays a critical role in the regulation of integrin *α*_IIb_ *β*_3_ affinity state and, therefore, controls platelet aggregation induced by soluble agonists [26, 27, 37, 40]. Important information on the mechanism of inside-out integrin activation has been collected using transfected cell lines to reconstitute this signaling pathway. These studies indicate that integrin signaling involves the interaction of the head domain of the cytoskeletal protein talin with specific sites of integrin *β*-tail and with the plasma membrane [41, 42]. Talin can synergize with kindlin-1 and -2 to mediate integrin *α*_IIb_ *β*_3_ conversion into the active state; however, the overexpression of kindlin alone is not sufficient to promote integrin activation [43]. In talin- and integrin *α*_IIb_ *β*_3_-expressing CHO cells, the coexpression of PKC at levels comparable to those present in platelets induces a strong responsiveness to PMA exposure, and the overexpression of a constitutive active form of Rap1a bypasses the requirement of PKC, indicating that, in this model, Rap1 lies downstream of PKC [44]. Among different potential Rap1 effectors, RIAM (Rap1-interacting adaptor molecule) was shown to be involved in integrin regulation, and its overexpression in cell lines bypasses the requirement of Rap1. Moreover, RIAM knockdown suppresses Rap1-mediated integrin activation, as well as the association between talin and integrin *β*-tail [45, 46]. However, the importance of RIAM in platelet physiology and integrin *α*_IIb_ *β*_3_-mediated platelet aggregation still needs to be determined and will require the development of RIAM knockout mouse models.

Further support to the hypothesis that Rap1b regulates integrin affinity was provided by the analysis of Rap1b and CalDAG-GEFI knockout mice. Generally, the lack of Rap1b or CalDAG-GEFI is coupled to a substantial defect in integrin *α*_IIb_ *β*_3_ activation and platelet aggregation. Rap1b-deficient platelets display a reduced aggregation in response to a wide range of concentrations of agonists, as ADP and epinephrine, and to low doses of collagen, thrombin receptor-specific agonist AYPGKF, and Ca^2+^ ionophore [37]. The lack of CalDAG-GEFI is associated with a more severe reduction of platelet aggregation caused by the same set of agonists, whereas the DAG analogue PMA and high doses of thrombin induce a comparable extent of platelet aggregation in wild-type and CalDAG-GEFI-deficient platelets. The CalDAG-GEFI-independent pathway leading to platelet aggregation requires PKC activation, subsequent granule release, and P2Y12 stimulation by secreted ADP [26, 28, 47].

Similarly to integrin *α*_IIb_ *β*_3_, also integrin *α*_2_ *β*_1_ is present on the platelet surface in at least two different affinity states for its ligands, and conversion into the high-affinity state facilitates collagen-mediated platelet activation [48–50]. In this context, it has been recently shown that, under certain conditions, Rap1b expression is required to allow a proper integrin *α*_2_ *β*_1_ activation. Indeed, platelets from Rap1b knockout mice show defective integrin *α*_2_ *β*_1_ activation upon stimulation of the collagen receptor GPVI, but not upon stimulation of thrombin or ADP receptors. However, the extent of adhesion to collagen via integrin *α*_2_ *β*_1_ is normal in platelets from Rap1b knockout mice, suggesting that adhesion to collagen under static conditions does not involve Rap1b-dependent stimulation of integrin *α*_2_ *β*_1_ [51].

Interestingly, in contrast to what was observed for Rap1b-deficient platelets, platelets from CalDAG-GEFI knockout mice display a reduced ability to adhere to immobilized collagen under static conditions, when compared to wild-type cells [27]. Moreover, also the *ex vivo* analysis of thrombus formation on immobilized collagen under flow has revealed a reduced area coverage and thrombus growth in CalDAG-GEFI knockout platelets. Interestingly, the addition of ADP and TxA_2_ increases the adhesion of CalDAG-GEFI knockout platelets to a collagen surface at low shear rates, without restoring thrombus growth [38]. In addition to the key role played in platelet adhesion to collagen, the expression of CalDAG-GEFI is also required for an efficient platelet interaction with other *β*_1_ integrin ligands as laminin and fibronectin [52].

The observed differences between Rap1b and CalDAG-GEFI knockout mice, which are particularly evident in terms of phenotype severity, may be related to possible additional functions of CalDAG-GEFI, independent of Rap1b activation, that can be required for efficient integrin-mediated adhesion. Moreover, in Rap1b knockout platelets Rap1a or Rap2b could partially compensate the lack of Rap1b. Indeed, although expressed at lower level Rap1a can be activated by platelet stimulation in Rap1b knockout platelets [51]. By contrast, since CalDAG-GEFI also stimulates Rap2b activity in platelets [53], this isoform cannot compensate the lack of Rap1b, and this may explain the more severe phenotype of CalDAG-GEFI knockout mice. However, it should also be considered that some discrepancy in the results obtained from CalDAG-GEFI and Rap1b knockout mice simply reflects the different experimental conditions adopted.

## 4. Rap1b Activation Downstream of Platelet Adhesion Receptors

An increasing number of observations indicate that Rap GTPases and adhesive receptors are connected in a bidirectional fashion. As described above, a major role for Rap GTPases is the regulation of integrin affinity for the specific ligands. In this context Rap1b activation is triggered by stimulation of many GPCRs in platelets and participates to the organization of the signaling pathway for integrin inside-out activation. However, in the last years, an increasing number of studies have proved the involvement of Rap1b also in integrin outside-in signaling and have documented its activation downstream of many platelet adhesive receptors.

### 4.1. Integrin *α*_**2**_ *β*_**1**_-Mediated Rap1b Activation

Integrin *α*_2_ *β*_1_ is a collagen receptor important for a proper hemostasis in humans, as documented by the observation that mutations of this receptor are associated with bleeding disorders and reduced platelet responses to collagen [54, 55]. Integrin *α*_2_ *β*_1_ is a critical platelet adhesion receptor, that interacts with collagen, by recognizing hexapeptidic sequences, such as GFOGER, GLOGER, GASGER, GROGER, and GLOGEN [56–58], but it also binds tenascin [59], and mediates platelet adhesion to the small proteoglycan decorin [60].

Platelet adhesion via integrin *α*_2_ *β*_1_ directly stimulates Rap1b activation, without the need for further autocrine stimulation by secreted ADP or released thromboxane A_2_ [27]. Experiments performed with platelets from genetically modified mice have shown that CalDAG-GEFI is the only GEF required for Rap1b stimulation downstream of integrin *α*_2_ *β*_1_ [27]. The dissection of the signaling pathway involved indicates that PLC*γ*2 expression and activation are mandatory for integrin *α*_2_ *β*_1_-triggered Rap1b activation. Interestingly, recruitment of platelet integrin *α*_2_ *β*_1_ mediates the activation of PLC*γ*2 by two redundant mechanisms: a classical Src-mediated phosphorylation of PLC*γ*2 itself and a phosphorylation-independent mechanism involving the Rac GTPase [61]. These observations indicate the existence of important crosstalk between Rac1 and Rap1b GTPases in the control of platelet adhesion and activation, with Rac1 being potentially upstream of Rap1b. As it will be discussed later, the existence of a crosstalk between Rap and Rac has been recently confirmed also downstream of GPVI [53].

Further analysis of CalDAG-GEFI and PLC*γ*2 knockout mice demonstrated that the lack of Rap1b activation is accompanied by a significant inhibition of integrin *α*_IIb_ *β*_3_ conversion into the high-affinity binding state for fibrinogen, demonstrating that Rap1b activation plays a key role in the crosstalk between integrins and regulates integrin *α*_2_ *β*_1_-mediated platelet aggregation [27, 61]. More recently, it has been demonstrated that maximal Rap1b activation induced by integrin *α*_2_ *β*_1_ downstream of PLC*γ*2 requires the contribution of an additional Ca^2+^-dependent signaling pathway involving the focal adhesion kinase Pyk2 and the subsequent stimulation of PI3K*β*. In fact, the lack or the impaired activation of Pyk2 and PI3K*β* causes a defective Rap1b activation triggered by integrin *α*_2_ *β*_1_ engagement [62]. These findings outline that, as previously observed in platelets stimulated with ADP or other soluble agonists [63–65], also in integrin *α*_2_ *β*_1_ outside-in signaling Rap1b activity is regulated by PI3K. Since virtually no residual Rap1b activity is detected in CalDAG-GEFI-deficient platelets, this observation points to a contribution of PI3K activity in the regulation of CalDAG-GEFI, but the molecular mechanism for this process is still to be defined.

### 4.2. GPVI-Mediated Rap Activation

GPVI is membrane glycoprotein specifically expressed in platelets, functionally associated with the ITAM-containing transmembrane adapter protein FcR *γ*-chain [66, 67]. It is well documented that platelet GPVI is responsible for the first set of signals induced by platelet interaction with collagen and that it strongly cooperates with integrin *α*_2_ *β*_1_ to mediate full collagen-induced response [50, 68]. The GPVI-FcR *γ*-chain complex initiates a tyrosine-kinase-based signaling cascade which involves Src and Syk kinases, the adaptor proteins LAT and SLP76, and leads to phosphorylation and stimulation of PLC*γ*2 [69]. Mouse or human platelets lacking GPVI–FcR *γ*-chain display severe defects in collagen-induced activation, integrin *α*_IIb_ *β*_3_ regulation and platelet aggregation [70–76].

As part of the signaling pathways for collagen-induced platelet activation, GPVI stimulation triggers Rap1b activation. This process, however, is at least partially dependent on ADP secretion and the subsequent stimulation of P2Y12 receptor, both in human and murine platelets [24, 65, 77]. Nevertheless, the existence of a direct, P2Y12-independent pathway of GPVI-mediated Rap1b activation has been confirmed by the analysis of aggregation of platelets collected from wild-type and Rap1b knockout mice, performed in the presence of ADP receptors antagonists. These experiments show that the lack of Rap1b is associated with a reduced ADP-independent, GPVI-mediated platelet aggregation, demonstrating that this GTPase is required for an efficient GPVI signaling [78]. Interestingly, direct Rap1b-activation-mediated downstream of GPVI depends on the activity of PI3K, and it has been demonstrated that the contribution of both the *α* and *β* isoforms of PI3K is required [65, 77, 78].

As for the other platelet collagen receptor, integrin *α*_2_ *β*_1_, CalDAG-GEFI is a specific regulator of Rap1b activation also downstream of GPVI [28, 53]. In addition, a number of GPVI-dependent responses have been found to be impaired in CalDAG-GEFI-deficient platelets. Some of these, such as integrin *α*_IIb_ *β*_3_ activation, and platelet aggregation are consistent with the well-documented role for Rap1b, and other defects, such as the reduction of ERK signaling leading to a decreased TxA_2_ synthesis, or the impaired granule secretion, point to possible novel implication for this GTPase in platelet function [28, 53, 79].

The GPVI-mediated signaling pathway leading to Rap1b activation involves the small GTPase Rac1 [53]. The observation that Rac1 is involved in PLC*γ*2-dependent stimulation of Rap1b downstream of integrin *α*_2_ *β*_1 _[61] has been extended in a recent work by Stefanini and coauthors showing that the two GTPases exert a mutual influence also downstream of GPVI. Indeed, Rap1b signaling sustains Rac1 activation, and, in turn, Rac1 provides a feedback regulation of Rap1 through CalDAG-GEFI and P2Y12.

Interestingly, the ability of GPVI-FcR *γ*-chain complex to stimulate Rap1b has been directly compared to the stimulation of the closely related Rap2b [24]. It has been reported that GPVI ligation results in a time-dependent Rap2b activation, that is not influenced by platelet aggregation (i.e., integrin *α*_IIb_ *β*_3_-mediated fibrinogen binding) and actin cytoskeleton remodelling. Differently to what was observed for Rap1b, secreted ADP plays only a negligible role in Rap2b activation triggered by GPVI, whereas Ca^2+^ mobilization and PKC activation are both required. Another remarkable difference between activation of Rap1b and Rap2b downstream of GPVI is that Rap2b stimulation is largely independent from PI3K activity. However, it is important to note that PI3K inhibitors suppress thrombin-induced Rap2b activation [24]. Rap1 and Rap2 are therefore differently regulated by PI3K, depending on the nature of the stimulus. Recently, it has been shown that Rap2b activation depends on CalDAG-GEFI and P2Y12 signaling, similarly to what was already demonstrated for Rap1b [53]. According to the lower sensitivity to GAPs, Rap2b displays a higher baseline activation that is also a more sustained in time, compared to that of Rap1b [53, 80]. Unfortunately, Rap2b knockout mice have not been generated yet. Therefore, our current information on the contribution of Rap2b to platelet adhesion is still really limited.

### 4.3. Rap1b Activation Mediated by Integrin *α*_**IIb**_ *β*_**3**_ Outside-In Signaling

Integrin *α*_IIb_ *β*_3_ (also known as GPIIb/IIIa) is the most abundant platelet membrane receptor and is responsible of the binding of platelets to soluble fibrinogen, a process that mediates platelet aggregation [2]. As introduced before, integrin *α*_IIb_ *β*_3_ undergoes a conformational change upon platelet activation, that increases the receptor affinity for fibrinogen. This inside-out activation of the integrin function involves Rap1b stimulation and its association with RIAM, talin and other signaling and cytoskeletal proteins, such as vinculin and kindlin [2, 44, 46]. However, in addition to the key role in platelet aggregation, integrin *α*_IIb_ *β*_3_ is also able to mediate platelet adhesion to immobilized fibrinogen and to other RGD-containing ligands, including VWF [81], vitronectin [82], fibronectin [83], and thrombospondin [84]. Integrin *α*_IIb_ *β*_3_ interaction with its ligands initiates an outside-in signaling pathway, that contributes to the regulation of the later phases of platelet activation and is required for firm platelet adhesion and spreading on extracellular matrices [85–87], fibrin clot retraction [88], platelet procoagulant activity, and microparticle release [89, 90].

The first evidence for the involvement of Rap1b in integrin *α*_IIb_ *β*_3_ outside-in signaling was obtained from studies with thrombin-stimulated platelets in the presence of integrin antagonists, including the peptide GRGDS, that prevent fibrinogen binding and platelet aggregation. In this context it was initially shown that sustained Rap1b activation mediated by thrombin requires the interaction between integrin *α*_IIb_ *β*_3_ and fibrinogen, as it was inhibited by the RGDS peptide [23].

The activation of Rap1b by integrin *α*_IIb_ *β*_3_ has been confirmed by the direct observation that platelet adhesion to immobilized fibrinogen stimulates the accumulation of GTP-bound Rap1b [61]. Integrin *α*_IIb_ *β*_3_-mediated Rap1b activation is regulated by multiple intracellular effectors, including Src kinases, PKC, and cytosolic Ca^2+^. Moreover, Rap1b-deficient platelets display a reduced spreading on fibrinogen compared with wild-type controls, whereas clot retraction is abolished, indicating that stimulation of Rap1b is important for integrin *α*_IIb_ *β*_3_-mediated platelet responses [37, 79].

### 4.4. GPIb-IX-V and VWF in Rap1b Activation

The GPIb-IX-V receptor complex, which contains four transmembrane proteins, GPIb*α*, GPIb*β*, GPIX, and GPV, mediates platelet binding to VWF in a shear-dependent fashion. The VWF-GPIb-IX-V interaction is required to slow down circulating platelets on the site of injury and is strongly involved in the regulation of integrin *α*_IIb_ *β*_3_ and in the formation of arterial thrombi [91]. The adhesive function of GPIb-IX-V is coupled to the generation of intracellular signals that support platelet activation mainly through the phosphorylation of the ITAM-bearing Fc*γ*IIA receptor, which is physically associated with it [92]. Moreover, Fc*γ*IIA receptor-independent signal transduction pathways occurring downstream of GPIb-IX-V and leading to protein phosphorylation, calcium oscillation, and integrin *α*_IIb_ *β*_3_ activation have been identified [93]. GPIb-IX-V ligation triggers the subsequent activation of integrin *α*_IIb_ *β*_3_ that mediates firm platelet adhesion and initiates thrombus formation.

Despite the relevance of the initial platelet adhesion through GPIb-IX-V at the site of arterial injury for the whole process of thrombus formation, the information about the ability of this adhesion receptor to trigger Rap GTPases activation is extremely limited. It has been shown that platelet stimulation with VWF triggers the activation of both Rap1b and Rap2b and promotes their association with the cell cytoskeleton through a process involving the Fc*γ*IIA receptor [23]. VWF-induced Rap2b translocation to the cytoskeleton, in particular, is dependent on integrin *α*_IIb_ *β*_3_ as it is prevented in the presence of anti-integrin *α*_IIb_ *β*_3_-specific antibodies, as well as in patients affected by Glanzmann's thrombasthenia, a genetic disorder associated with the lack of expression of integrin *α*_IIb_ *β*_3_ [22].

Antibody-mediated clustering of Fc*γ*IIA receptor, that induces its tyrosine phosphorylation and mimic the signaling pathway triggered by VWF-mediated GPIb-IX-V stimulation, is coupled to Rap1b activation in a fashion completely dependent on secreted ADP [94]. Unfortunately, studies that used transgenic mouse models to elucidate the role of Rap1b in GPIb-IX-V-mediated platelet responses have not been reported.

## 5. Conclusions

**Figure 1:**
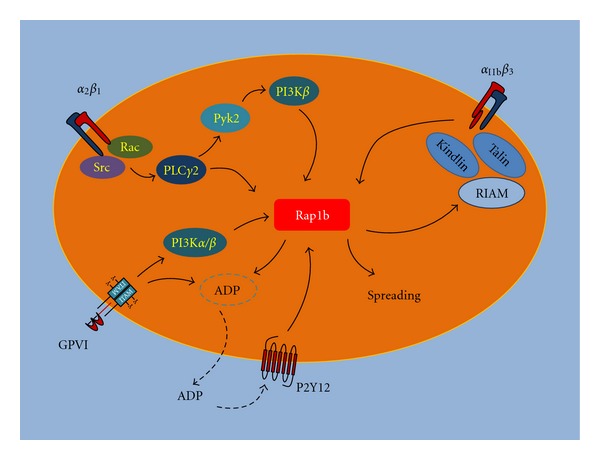
Schematic diagram of the main pathways regulating Rap1b activation during platelet adhesion. The figure summarizes the major players involved in Rap1b activation mediated by platelet adhesion receptors. Downstream of integrin *α*_2_ *β*_1_, PLC*γ*2 plays a critical role for Rap1b activation, which, however, requires also the Pyk2-mediated stimulation of PI3K*β*. Rap1 stimulates inside-out activation on integrin *α*_IIb_ *β*_3_ and thus it is a central player in the crosstalk between these two integrin receptors. The collagen receptor GPVI stimulates Rap1b both directly, through PI3K*α* and *β*, and indirectly through the autocrine stimulation of the P2Y12 receptor by secreted ADP. Moreover, activated Rap1b facilitates platelet granule secretion and ADP release. Rap1b-mediated inside-out activation of integrin *α*_IIb_ *β*_3_ involves the Rap1 effector RIAM and the cytoskeletal proteins talin and kindlin. In turn, integrin *α*_IIb_ *β*_3_ binding to fibrinogen stimulates an outside-in signaling able to promote Rap1b activation, which is an essential step for platelet spreading on fibrinogen.

The great effort devoted to understand the roles of Rap GTPases in the regulation of platelet function produced a huge amount of evidence demonstrating their critical role in haemostasis and thrombosis. As schematically summarized in Figure 1, the involvement of Rap GTPases in adhesion dynamics is complex and bidirectional, as they are both activated by adhesive receptors and essential for the regulation of the adhesive properties of integrins. The crucial implication of Rap GTPases in the control of cell adhesion suggests that the identification of their effectors may help to define novel possible targets for effective antiplatelet therapies for the treatment of cardiovascular diseases.
